# Exploring *Saduria entomon* (Crustacea Isopoda) as a New Source for Chitin and Chitosan Isolation

**DOI:** 10.3390/ijms232416125

**Published:** 2022-12-17

**Authors:** Isabel Rodriguez-Veiga, Niuris Acosta, Inmaculada Aranaz, Aldona Dobrzycka-Krahel

**Affiliations:** 1 Instituto Pluridisciplinar, Universidad Complutense de Madrid, Paseo Juan XXIII, Num. 1, E-28040 Madrid, Spain; 2 Departamento de Química en Ciencias Farmacéuticas, Facultad de Farmacia, Universidad Complutense de Madrid, Plaza de Ramón y Cajal s/n, E-28040 Madrid, Spain; 3 Faculty of Oceanography and Geography, Institute of Oceanography, Department of Experimental Ecology of Marine Organisms, University of Gdansk, Al. Marszałka Piłsudskiego 46, 81-378 Gdynia, Poland; 4 Business Faculty, WSB University in Gdańsk, Al. Grunwaldzka 238A, 80-266 Gdańsk, Poland

**Keywords:** antimicrobial, antioxidant, polymer isolation, chitin, chitosan

## Abstract

Chitin and chitosan demand is growing very fast due to interest from industries such as pharmaceutical, cosmetic, agricultural and others. New sources for chitin and chitosan isolation are being extensively searched to fulfil this demand. In this paper, *Saduria entomon* a Baltic benthic crustacean, is evaluated as a source for chitin and chitosan isolation. Chitin and chitosan yield from *S. entomon* were 14.8 and 8.2%, respectively, in a similar range to other sources. Samples were characterized in terms of physicochemical properties (acetylation degree, molecular weight, thermal stability, and crystallinity) and two biological properties, antimicrobial activity and antioxidant activity were evaluated. Chitosan *S. entomon* exhibited antimicrobial activity against *S. aureus* but not against *E. coli.* An antioxidant activity of 20.98 TROLOX µmol equivalent/g polymer was detected for the chitosan sample. These properties are very promising for the use of this organism as a source for chitin and chitosan isolation in the biomedical field.

## 1. Introduction

Chitin and chitosan are valuable biomaterials because of their biological properties, such as biocompatibility, non-toxicity, biodegradability, antioxidant, and antimicrobial activities [1,2,3]. Chitin is a constituent of different crustaceans, such as crabs, shrimps, crayfish, and others [4,5,6]. Chitosan, the deacetylated derived of chitin, is especially attractive for the pharmaceutical industry because of its many possible applications, taking advantage of both its technological and biological properties, such as antioxidant and antimicrobial effects [7,8,9]. Recognition of a biomaterial’s properties is important for planning possible applications. Moreover, the natural environment of a crustacean can explain the properties of polymers that are components of its carapax.

The object of this study was *Saduria entomon*, a benthic crustacean species which is a glacial relict of the brackish Baltic Sea where it is collected. Its distribution is restricted to the northern hemisphere, in the Baltic, Caspian, Aral and Arctic Seas [10,11,12]. This species also occurs in several lakes of the Scandinavian region: Mälaren, Vättern, Vänern, Mjörn and Ladoga Lakes [13]. *S. entomon* is one of the biggest Baltic crustaceans, and grows to about 8 cm long [14]. It is a stenothermal crustacean, preferring water temperatures from 2 to 12 °C [15] but it can survive at temperatures of 15 to 20 °C [14]. It is an euryhaline species living in the Baltic Sea in a salinity range of about 1 to 20 PSU [14]. It feeds on benthic invertebrates, mostly amphipod *Monoporeia affinis* [14,16,17], and also plays an important role in the ecosystem. Being a scavenger, it contributes to the removal of dead organisms from the seabed [18] and it is an important component of the Baltic Sea zoobenthos [19] where it is buried into the surface of the bottom [20]. It has been object of many studies on distribution and status [21] and has also been explored in ecophysiological studies [22,23,24].

Previous analysis of the biochemical composition of this species carried out by Normant and Szaniawska [18] showed that the average proportions of compounds found it contained were as follows: 28.2% proteins, 6.7% lipids, 8.0% carbohydrates and 28.0% ash; therefore, proteins are the most abundant compounds in *S. entomon*. Since this species is an important food source for many commercially used fish species e.g., for cod *Gadus morhua*, flounder *Platichthyes flesus luscus* or turbot *Psetta maxima maeotica* [16], it is a potential source of protein in aquaculture for fish food.

The aim of the paper was to evaluate, for the first time, *S entomon* as a source for chitin and chitosan production, and to characterize its main physicochemical and biological properties to determine its suitability for polymer isolation for biomedical purposes.

## 2. Results

### 2.1. Chitin and Chitosan Isolation

**Scheme 1:**
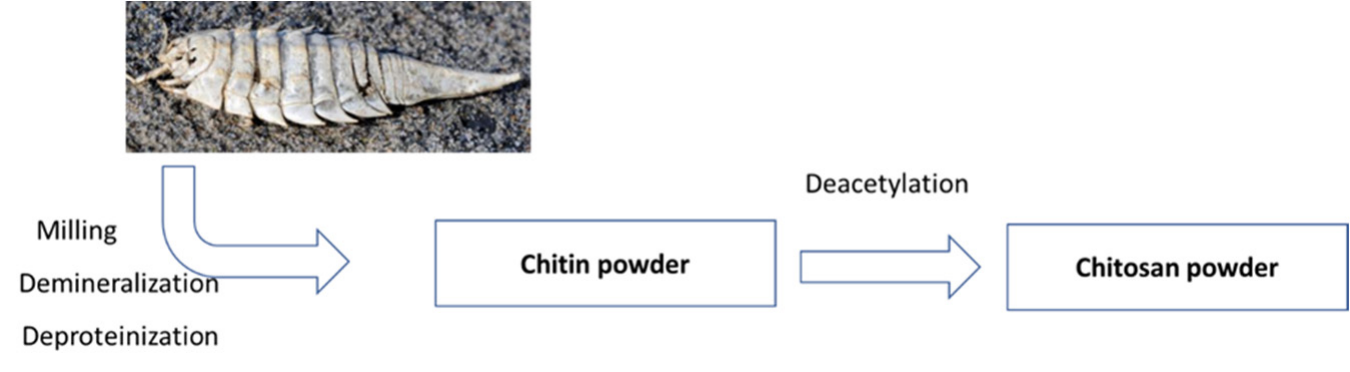
Isolation of chitin and chitosan production from *S. entomon*.

Chitin isolation was carried out in two steps and produced by chemical deacetylation (Scheme 1).

The percentage of chitin content in the crustacean’s body mass depends on the source and isolation method and is usually from 2 to 12% (*w*/*w*) [25]. The amount of chitin isolated from *S. entomon* exoskeleton was 14.8% (*w*/*w*). After deacetylation, chitosan was produced from this chitin with a yield of 64.4% (*w*/*w*). Therefore, chitosan yield from the *S. entomon* body was 8.2% (*w*/*w*). The chitin sample was brownish, while a white chitosan was produced without any discolouration treatment that could alter the polymer properties; for example, by reducing its molecular weight or oxidizing the polymer.

### 2.2. Chitin and Chitosan Physicochemical Characterization

Elementary analysis was carried out to determine the chemical composition of the polymer samples (acetylation degree). The percentage of N in fully acetylated chitin was 6.9% and in fully deacetylated chitosan was 8.9%. Therefore, it can be concluded that the higher the percent N content the lower the acetylation degree. In Table 1, it is shown that our results followed this trend, with a higher N content in chitosan than in chitin (6.58% vs. 6.41%). However, it seems that nitrogen content was overestimated, since DA calculated by Equation (5) gave a value of 129% for chitin and 127% for chitosan.

**Figure 1:**
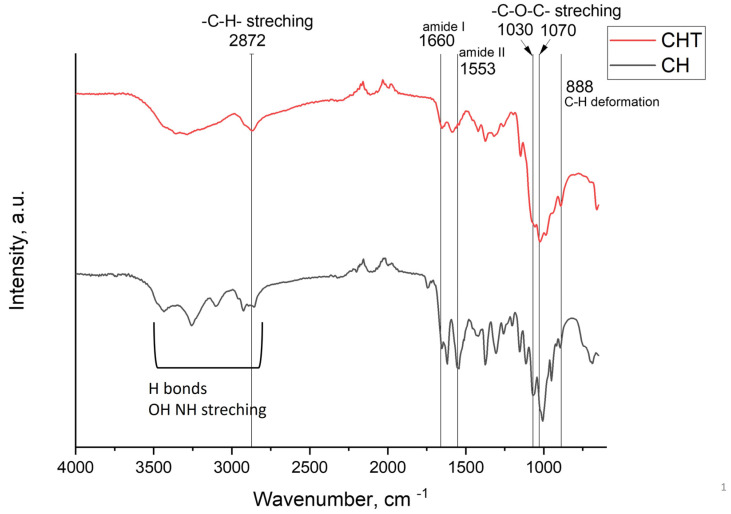
FTIR spectra of isolated chitin (CH) and chitosan samples (CHT).

The ATR-FTIR spectra of isolated chitin and chitosan samples are shown in Figure 1. The main bands observed by ATR-FTIR spectroscopy were assigned according to the literature [26,27].

Typical bands from chitin and chitosan, among other bands, were observed (Figure 1) The chitin amide I band was observed around 1660 cm^−1^, in good agreement with the literature. In this spectrum, we didn’t observe the typical doublet of alpha chitin due to the low resolution of ATR spectrum when compared to standard transmission spectrum from KBr pellets [26]. The amide II band appeared around 1553 cm^−1^. The CH deformation of the β-glycosidic bond appeared around 888 cm^−1^. Several bands in the region 1200–950 cm^−1^ were due to C-O-C and C-O vibration regions. In the region 3500–2500 cm^−1^, bands due to OH and NH stretching were also observed. The chitosan spectra showed fewer bands than the chitin spectra, one in particular in the region 1800–900 cm^−1^. In the region 3500–3000 cm^−1^, a wide band was observed due to intramolecular hydrogen bonds, and O-H and N-H stretching bands overlapped. A single band around 2872 cm^−1^ due to C-H stretching was observed. An amide II band (C-O stretching of acetyl groups) was observed around 1661 cm^−1^ indicating the presence of residual acetyl groups.

Successful chitin deacetylation to produce chitosan was further corroborated by a solubility assay in HCl 0.1M, where more than 99% of the polymer (10 mg/mL) was solubilized, indicating the presence of large number of primary amino groups on the polymer structure, and by the determination of chitosan deacetylation degree by UV-spectroscopy method [28]. By using this method, chitosan acetylation degree was determined to be 8% in good agreement with the presence of residual N-acetyl groups observed in ATR-FTIR analysis (band at 1661 cm^−1^).

The chitosan sample exhibited an intrinsic viscosity in acetic/acetate buffer of 6.2 dL/g, and the sample in solution was easy to handle. From the intrinsic viscosity, the viscosity molecular weight estimated by Mark–Houwink–Sakurada equation, an Mv of 160 kDa was calculated [29]. Chitosan molecular weight of 313 kDa and polydispersity of 1.56 were also estimated from Gel Permeation Chromatography (GPC). These data demonstrated that medium molecular weight chitosan with moderate polydispersity can be isolated from *S. entomon*.

**Figure 2:**
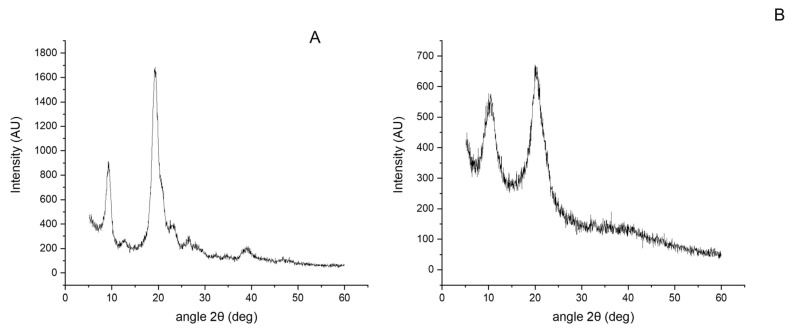
XRD pattern of chitin (**A**) and chitosan (**B**) isolated from *Saduria entomon*.

In Figure 2, the XRD spectra of the samples are shown. The chitin sample was strongly crystalline with a crystalline degree of 87.7%. Chitin crystallinity from alpha chitin samples varies depending on the source. Cardenas et al. found values of 80.6, 81.9, 82.7, and 76.2% for king crab chitin, crab chitin, lobster chitin and shrimp chitin, respectively [30]. On the contrary, chitosan was less crystalline with a degree of crystallinity of 62%. This result is in good agreement with previous work that showed a reduction of crystalline index as the deacetylation degree increased [31].

In the chitin sample, two main reflections at 9.26 and 19.46° were observed, which correspond with Miller planes (020) and (040)/(110). Other small reflections belonged to planes 101, 130 and 013 according to the literature [30]. The XRD spectrum of chitosan also showed main reflections at 10.4 and 20.3. and a broad band between 30–50 due to the presence of amorphous regions [32].

**Figure 3:**
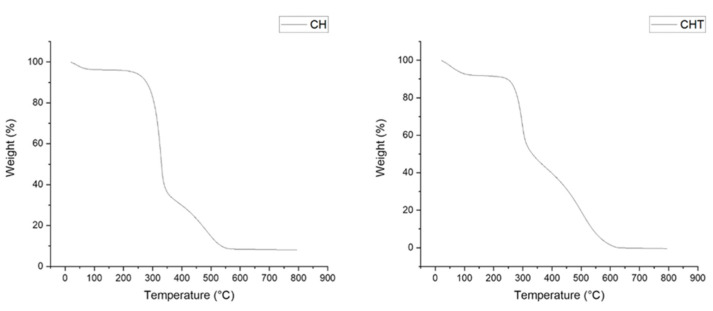
Thermal analysis of chitin and chitosan samples.

The thermal analysis of chitin and chitosan is shown in Figure 3. Chitin and chitosan thermographs exhibited three decomposition steps, the first one around 95 °C for chitin, and 100 °C for chitosan, corresponded to water evaporation. The process led to a mass weight loss of 4% and 8% for chitin and chitosan, respectively. A second peak between 200 and 380 °C for chitin, and between 225 and 370 °C for chitosan, was also observed. This loss was due to the dehydration of the saccharide backbone, the polymerization of the degradation products, and the decomposition of the acetyl group [33]. Finally, a third decomposition was observed between 400 and 600 °C.

**Figure 4:**
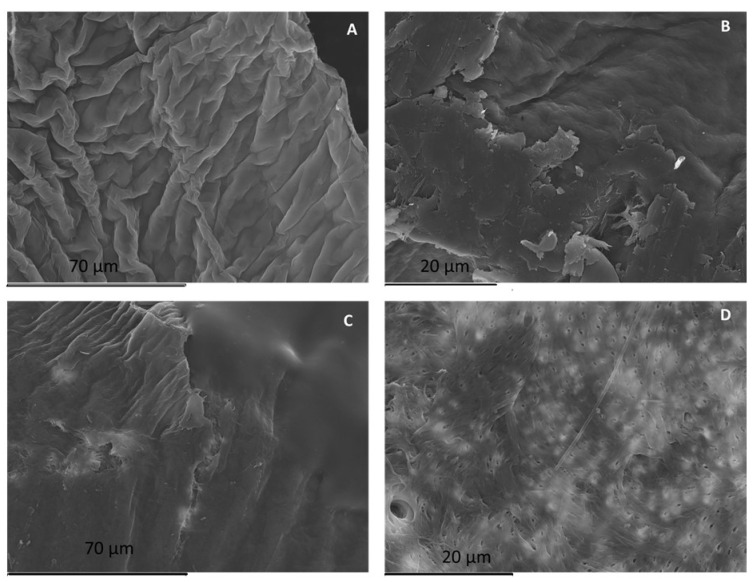
SEM images of chitin (**A**,**B**) and chitosan (**C**,**D**). Bar 70 µm in left panel and 20 µm in right panel.

The morphology of chitin is shown in Figure 4A,B. A rough surface without pores were observed as seen in Figure 4C,D, while chitosan morphology exhibited a mixture of non-porous and porous structure.

### 2.3. Antioxidant Activity

Chitin, and by extension, chitosan, exhibit a large number of biological activities such as antimicrobial, antioxidant, anti-inflammatory and mucoadhesive effects [34,35].

In this work, we evaluated two of these biological properties, i.e., antioxidant and antimicrobial activities. The antioxidant activity of chitosan in solution was determined using TROLOX as the gold standard. Our results showed an antioxidant activity of 20.98 TROLOX µmol equivalent/g polymer. In previous work, Mengibar el al. found values between 9 and 22 TROLOX µmol equivalent/g polymer depending on chitosan molecular weight [36]. Similar results regarding the effect of chitosan MW were observed by Kim and Thomas although we cannot compare numerically the results since they were expressed as scavenging percentages [37].

Chitin, on the other hand, did not exhibit antioxidant activity in our assay which is in good agreement with the possible scavenging mechanism. It has been reported that antioxidant activity is related to the presence of the hydroxyl group (C6) and the amino group (C2) so that active hydroxyl and amino groups can react with free radicals. In chitin, almost no amino groups are present in the structure, and due to its lack of solubility, the accessibility of hydroxyl groups to ROS is low [38].

### 2.4. Antimicrobial Activity

The antimicrobial activity of both chitin and chitosan was tested against *Escherichia coli* as a Gram-negative and *Staphylococcus aureus* as a Gram-positive model. The antimicrobial behavior of chitosan is related to many parameters, such as pH, chitosan characteristics (MW and DD), bacterial strain, physical state, and experimental methods. Therefore, the antimicrobial activity was tested in powder and solution, and the antimicrobial activities of crab chitosan and lobster chitosan were studied for comparative purposes.

**Figure 5:**
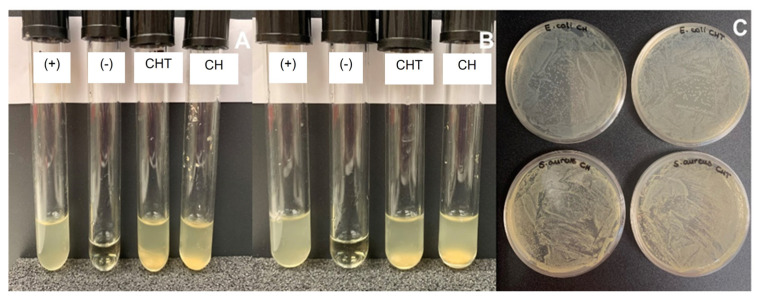
Antimicrobial activity assay of chitin (CH) and chitosan (CHT) powder from *S. entomon* against *E. coli* (**A**) and *S. aureus* (**B**). Growth on plates (**C**).

In a first set of experiments the antimicrobial activity of the polymers as powders was determined. To this end, suspensions of 10 mg/mL of each polymer (chitin or chitosan) and source (*S. entomon*, crab or lobster) were tested against the two types of bacteria. For *S. entomon*, in all cases turbidity was observed in the tubes (Figure 5A, B). In order to determine if this turbidity was due to bacterial growth or to the polymers themselves, 100 µL of each solution from tube were poured onto agar Mueller Hinton plates and the plates were incubated at 37 °C for 24 h. To determine whether the antimicrobial activity of the polymer was effective, the criterion was that growth should be less than or equal to 0.001% (the inoculum being 100%, which is equivalent to 500,000 CFU (colony-forming units)). As seen in Figure 5C and in Table 2, neither chitin nor chitosan as powders exhibited antimicrobial activity against *E. coli* or *S. aureus*.

**Figure 6:**
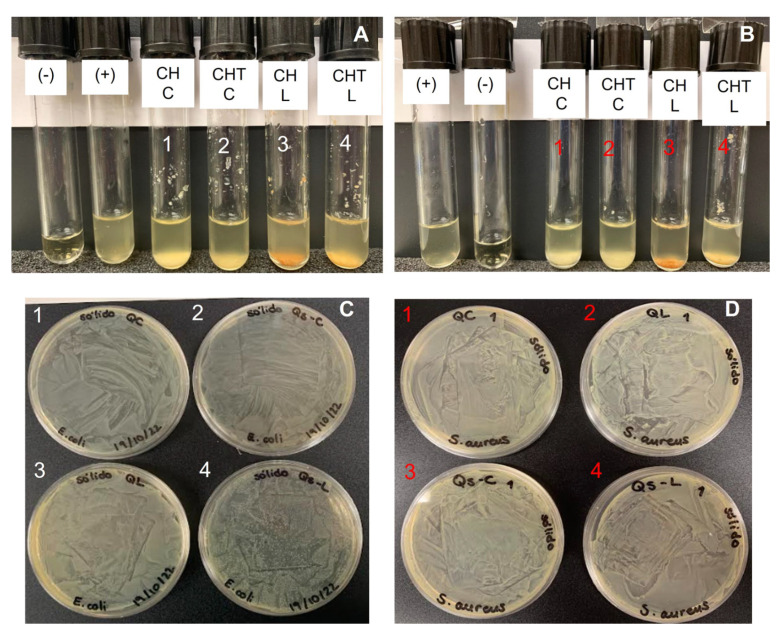
Antimicrobial activity of chitin (CH) and chitosan (CHT) powder from crab (C) and lobster (L) against *E. coli* (**A**) and *S. aureus* (**B**). Growth on plates of antimicrobial assay tubes of *E. coli* (**C**) and *S. aureus* (**D**). Tube (+) is positive control and tube (–) is negative control.

As shown in Figure 6, similar results were observed when the antimicrobial activity of solid suspensions of crab and lobster chitin and chitosan were tested against *E. coli* and *S. aureus*. Again, turbidity appeared in the tubes (Figure 6A,B) and bacterial growth was clearly shown in the plates (Figure 6C,D). As shown in Table 2 bacterial growth was higher than 99.9% and, therefore, it can be concluded that chitin and chitosan powders did not exhibit antimicrobial activities against the tested bacteria.

In a second set of experiments, the antimicrobial activity of chitosan in solution against *E. coli* and *S. aureus* was determined. To this end, the polymers were dissolved in AcOH 0.15 mol/L ammonium acetate/0.2 mol/L acetic acid buffer at a final concentration of 2% *w*/*v* in the case of *S. entomon* and lobster chitosan, and at 1% *w*/*v* for crab chitosan, since at higher concentrations the sample was difficult to handle.

**Figure 7:**
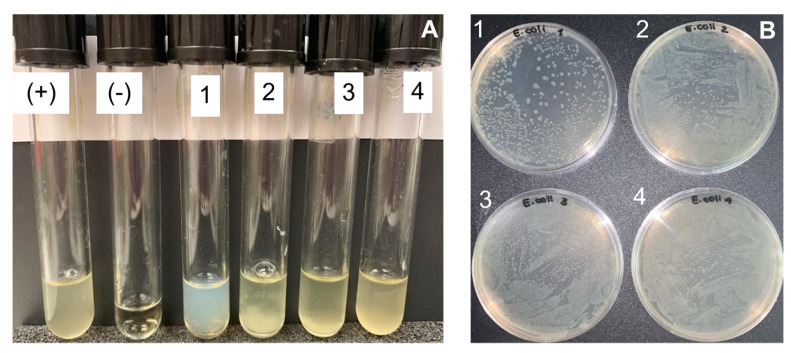
Antimicrobial activity of *S. entomon* chitosan solution in a broth macrodilution assay against *E. coli* (**A**). Growth on plates (**B**). Tube 1: 3.34 mg/mL, tube 2: 1.11 mg/mL, tube 3: 0.37 mg/mL and tube 4 to 0.125 mg/mL. Tube (+) is positive control and tube (–) is a negative control.

When *S. entomon* chitosan was tested against *E. coli* (up to 3.34 mg/mL), turbidity appeared in all tubes and bacterial growth was confirmed on all plates (Figure 7). In tube 1, less bacterial growth was observed than in the other ones and it was possible to determine a value for CFU due to the presence of single colonies rather than a lawn (Table 3).

Although chitosan from *S. entomon* did not exhibit antimicrobial activity against *E. coli*, at least up to 3.34 mg/mL, previous studies have shown that chitosan exhibits antimicrobial activity against *E. coli* with a large variety of MIC values depending on molecular weight (from 0.05 to 0.2% *w*/*v* at 50, 3 and 1000 kDa) [39]. Other authors have reported even lower values, such as Seo et al. who found the MIC of chitosan for *E. coli* was 0.002% (*w*/*v*) [40]. Uchida et al. reported the MIC to be 0.025% (*w*/*v*) [41]. Jeon et al. reported that MIC values of chitosan were less than or equal to 0.06% (*w*/*v*) against Gram-negative and Gram-positive bacteria [42]. For crab chitosan, the MIC against *E. Coli* was determined to be 1.2 mg/mL in acetic acid 1% *v*/*v* [43].

**Figure 8:**
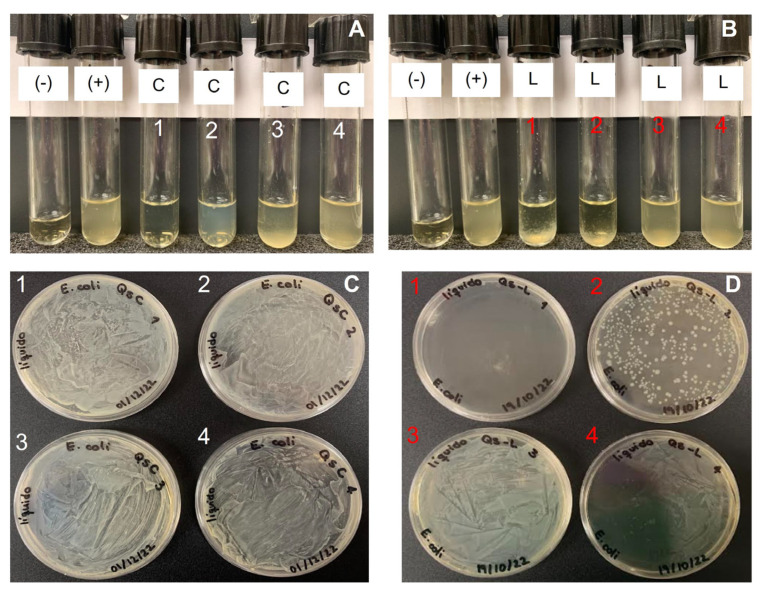
Antimicrobial activity of crab chitosan (crab chitosan: C) in a broth macrodilution assay against *E. coli* (**A**). Antimicrobial activity of lobster chitosan (lobster chitosan: L) in a broth macrodilution assay against *E. coli* (**B**). Growth on plates of crab chitosan assay (**C**) and lobster chitosan assay (**D**). In crab chitosan tube 1: 1.5 mg/mL, tube 2: 0.375 mg/mL, tube 3: 0.1 mg/mL and tube 4: 0.02 mg/mL. In lobster chitosan, concentrations for tube 1 to 4 were 3.34 mg/mL, 1.11 mg/mL, 0.37 mg/mL and 0.125 mg/mL respectively.

**Figure 9:**
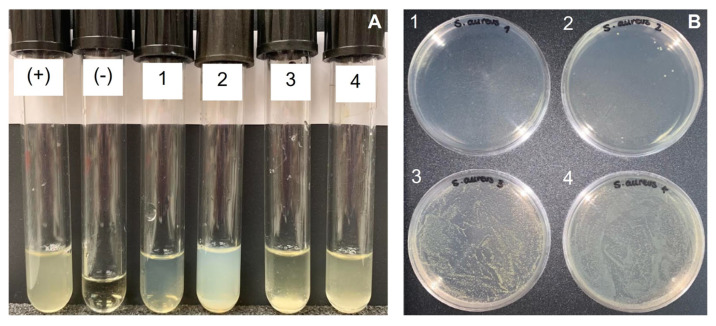
Antimicrobial activity of *S. entomon* chitosan in a broth macrodilution assay against *S. aureus* (**A**). Growth on plates (**B**). Tube 1: 3.34 mg/mL, tube 2: 1.11 mg/mL, tube 3:0.37 mg/mL and tube 4: 0.125 mg/mL.

To ascertain that the lack of activity against *E. coli* was not due to our experimental conditions, we tested a chitosan sample from crab and another from lobster (Figure 8A). After a naked-eye examination, crab chitosan inhibited bacterial growth at 1.5 mg/mL, since no turbidity was observed in tube 1. However, growth on agar Mueller Hinton plates was observed in all tubes (Figure 8C). This can be explained by a bacteriostatic effect of crab chitosan in *E. coli* instead of a bactericidal effect. In this way, bacteria show a “dormant” phenotype in the presence of a certain concentration of the antimicrobial agent, but once they are plated in the absence of chitosan, bacteria can grow. On the other hand, lobster chitosan showed inhibition of *E. coli* growth, with precipitate in tubes 1 and 2 (Figure 9B). However, no growth was observed in the plate corresponding to Tube 1 (3.34 mg/mL), and bacterial growth was partially inhibited in tube 2 (Figure 8D, Table 3). Hence, MIC against *E. coli* was determined to be near 3.34 mg/mL for lobster chitosan.

MIC for different *S. aureus* strains ranged from 1 to 16 mg/mL when low MW chitosan and chitooligosaccharides samples were tested [44]. For crab chitosan, MIC against *S. aureus* was determined to be 1.3 mg/mL in acetic acid 1% *v*/*v* [43]. Results for the antimicrobial assay for *S. entomon* against *S. aureus* are shown in Figure 9. Turbidity was observed in *S. entomon* tubes, but it corresponded to the presence of the polymer, since no growth was observed in the plate corresponding to tube 1 (3.34 mg/mL), and almost no growth was observed in the plate corresponding to tube 2 (1.11 mg/mL) where only seven CFU were counted, corresponding to an inhibiton of around 99.999%. Therefore, MIC against *S. aureus* was determined to be around 3.34 mg/mL, since at this concentration bacterial growth was completely inhibited.

**Figure 10:**
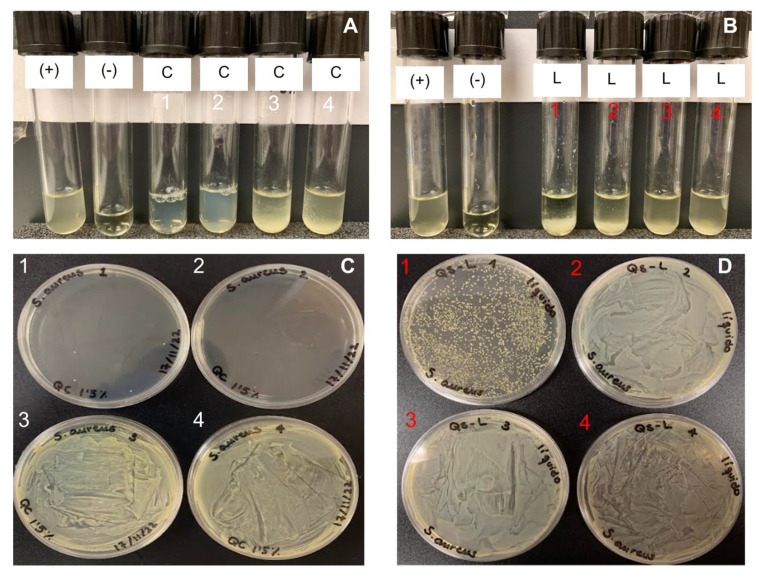
Antimicrobial activity of crab chitosan (C) in a broth macrodilution assay against *S. aureus* (**A**). Antimicrobial activity of lobster chitosan (L) in a broth macrodilution assay against *E. coli* (**B**). Growth on plates of crab chitosan assay (**C**) and lobster chitosan assay (**D**). In crab chitosan tube 1: 1.5 mg/mL, tube 2: 0.375 mg/mL, tube 3: 0.1 mg/mL and tube 4: 0.02 mg/mL. In lobster chitosan, concentrations for tube 1 to 4 were 3.34 mg/mL, 1.11 mg/mL, 0.37 mg/mL and 0.125 mg/mL respectively.

In crab chitosan, strong antimicrobial activity was observed with no growth on the plate corresponding to Tube 2, containing a concentration of the polymer of 0.375 mg/mL (Figure 10A,C). However, we cannot properly speak of MIC at this concentration since some colonies grew on the plate corresponding to tube 1 (MIC is defined as the concentration at which antibacterial agent completely prevents visible growth).

Lobster chitosan did not show an antimicrobial effect against *S. aureus (*Figure 10B), despite showing moderate growth on the agar plate corresponding to tube 1, and the concentration of microorganisms was too high to consider a relevant antimicrobial effect (Figure 10D, Table 3). This effect can be explained, once again, due to the loss of a bacteriostatic effect of chitosan when bacteria are plated in a non-restrictive medium.

As seen in Table 3, chitosan from *S. entomon* and crab showed better performance against *S. aureus* than *E. coli*, in good agreement with previous results that showed more effective suppression against Gram-positive bacteria comparing to Gram-negative bacteria when chitosan and chitooligosaccharides were evaluated [42].

## 3. Discussion

Chitin and chitosan are polymers of interest in different fields such as pharmacy, biomedicine, health care, agriculture, the food industry and wastewater treatment, among others, due to their interesting technological and biological properties [34,35,45]. Most commercial chitin and chitosan samples are isolated from crustacean shells from Asia, while production in Europe (e.g., Primex, Heppe Medical) is quite limited, even though the European market is a top client [46]. There is a growing interest in finding new sources for chitin and chitosan that avoid dependence on Asian markets. In this paper, we evaluate the exoskeleton of *S entomon* as a possible source of chitin and chitosan. To our knowledge, this is the first time that chitin and chitosan have been isolated and characterized from this source.

Chitin was isolated following the standard two step methodology (Scheme 1), and chitosan was produced by chemical deacetylation of chitin. The *S. entomon* chitin yield was slightly higher than typical values observed from other chitin sources [25]. This could be due to the presence of some impurities as revealed by elementary analysis (overestimation of DA content) and FTIR-ATR (presence of large number of bands). The overestimation of DA by elementary analysis has been previously reported by other authors. Chitin samples isolated from *Dociostaurus maroccanus* showed DA values of 232%, *Ogmocnemis asellus* values of 169%, and *Vespa crabro* values of 127%. [47,48]. These authors have found that chitosan calculations, in general, were more accurate. They suggested that the overestimation is due to the presence of nitrogen-free impurities such as lipids and sugars in the chitin samples that are removed in the conversion of chitin to chitosan. However, in the case of *S. entomon*, it seems that these impurities were present both in chitin and chitosan samples and, therefore, elementary analysis is not a suitable technique to determine the acetylation degree of the samples from this source.

Crystallinity, morphology, and thermal stability were similar to those previously reported for these kinds of polymers. *S. entomon* chitosan showed an acetylation degree of 8%, a medium molecular weight, and moderate polydispersity index. The sample also showed moderate viscosity which is of interest for proper handling.

Antioxidant activity of chitin and chitosan was evaluated. In previous work, Mengibar et al. found values between 9 and 22 Trolox µmol equivalent/g polymer depending on the chitosan molecular weight [36]. Our results were slightly higher than those reported by Mengibar et al. for chitosan isolated from *S. entomon*. Antioxidants neutralize the free radicals in biological cells and are associated with a lower risk of oxidative stress-related diseases such as cardiovascular diseases, cancer, and other diseases, and have applications in different fields, as may be in the case of chitosan obtained from *S. entomon* [49]. Moreover, antioxidant activity is also related to wound healing applications. When the skin is damaged, large amounts of oxygen-containing reactive species (ROS) are produced, disrupting the wound healing process. This is due to biological damage by degradation of lipids, proteins, nucleic acids and, ultimately, cell death. Therefore, molecules with antioxidant activity are postulated to help control wound oxidative stress and thus accelerating wound healing [50].

The antimicrobial activity of both chitin and chitosan was tested again *E coli* and *S. aureus. E coli* strains are frequently isolated from skin and soft tissue infections, and these strains exhibit a similar virulence to those isolated from urinary tract infections and bacteremia [51]. *S. aureus* can cause a large variety of skin infections, including pimples, impetigo, boils, cellulitis folliculitis, carbuncles, scalded skin syndrome, and abscesses, among other infections [52]. The antimicrobial behavior of chitosan is related to a large number of parameters such as pH, chitosan characteristics (MW and DD), bacterial strain and experimental methods. Therefore, the antimicrobial activity of crab chitosan and lobster chitosan was studied for comparative purposes.

Chitin samples (*S. entomon*, lobster, crab) exhibited antimicrobial activity against the bacterial strains tested. On the other hand, chitosan from *S. entomon* did not exhibit activity against *E. coli*, but a moderate effect against *S. aureus* was observed. It is remarkable that neither crab chitosan nor lobster chitosan exhibited activity against *E. coli*, and lobster chitosan did not have activity against *S. aureus* either. So, *S. entomon* seems to have better performance against *S. aureus* than other crustacean chitosans under our assay conditions.

Antioxidant activities and antimicrobial activities are important properties for biomaterials to be used in wound healing processes [53,54,55]. Chitosan from *S. entomon* showed good antioxidant and antimicrobial activity, at least against *S. aureus.* These results point to a possible use of this source in wound healing applications. However other properties described for chitosan, such as anti-inflammatory [56] and haemostatic activities [57], must be studied to gain more insight into the role of this in wound healing. Moreover, antimicrobial studies against other strains are also highly recommended.

## 4. Materials and Methods

### 4.1. Preparation of the Material for Further Analysis

Samples of *S. entomon* were collected from the Gulf of Gdansk (southern Baltic Sea) on 15th November 2018. After visual inspection, plastics, nets and sand were discarded Animals were washed with water and boiled in water for 8 h. The water was changed five times, and after this, the animals´ exoskeletons were dried in an oven at 50 °C for 24 h. Finally, the sample was milled with a coffee grinder (Moulinex, Barcelona, Spain) and the sample was passed through an 18-mesh sieve (1 mm).

### 4.2. Chitin and Chitosan Extraction Process

Chitin isolation was carried out in two steps from crustacean sources. First, a demineralization process was carried out with a solution of HCl, (2 M, ratio dry material: acid 1:10) at room temperature under stirring for 2 h. The demineralized samples were exhaustively washed with water to remove acid. Secondly, deproteinization was carried out with an NaOH solution (2% *w*/*v*, ratio dry material: basic solution 1:10) at 80 °C under mechanical stirring for 30 min. The samples were exhaustively washed with water to remove NaOH and then dried. The isolated chitin was further deacetylated to produce chitosan using a 70% *w*/*v* NaOH solution (ratio dry material: base 1:20) at 90–100 °C for 4 h under mechanical stirring. The sample was thoroughly washed with water until a neutral pH was observed. Finally, chitosan was dried in an oven at 45 °C overnight.

Chitin and chitosan yields were determined according to Equations (1)–(3).

(1) $$Chitin\;yield\;\left(\%\right)=\frac{weight\;of\;chitin}{weight\;of\;crustacean\;shell}*100$$

(2) $$Chitosan\;yield\;from\;chitin\;\left(\%\right)=\frac{weight\;of\;chitosan}{weight\;of\;chitin}*100$$

(3) $$Chitosan\;yield\;from\;crustacean\;shell\;\left(\%\right)=\frac{weight\;of\;chitosan}{weight\;of\;crustacean\;shell}*100$$

### 4.3. Solubility Test

Chitosan solubility in an acidic medium (HCl 0.1M, pH 1) was tested. Briefly, 20 mg of the polymer was added to 2 mL of HCl solution, and the solution was stirred overnight. After that, the sample was filtered through a 5 µm filter and the filter was dried at 50 °C. The amount of non-solubilized polymer was determined gravimetrically by weighting the dry filter before and after the filtration process.

(4) $$Solubility\;\left(\%\right)=\frac{amount\;of\;insoluble\;sample}{polymer\;weight}*100$$

### 4.4. Elementary Analysis

The C, N, H composition of the samples was determined using an elemental microanalyzer LECO CHNS 932 3288 (LECO, St. Joseph, MI, USA). The acetylation degree of the chitin and chitosan samples was determined according to Equation (5) [58].

(5) $$DA=\frac{\frac{C}{N}-5.14}{1.72}*100$$

### 4.5. Determination of Acetylation Degree by UV-vis Analysis

The chitosan acetylation degree was determined by the UV-VIS spectrophotometric (analytik Jena GmbH, Jena, Germany) method of the first derivative as described by Muzzarelli [28]. N-acetylglucosamine was used as the standard for the calibration curve.

### 4.6. ATR-FTIR Spectra

Chitin and chitosan samples were analyzed in an Agilent Technologies Cary 630 FTIR (Agilent, Santa Clara, USA). The spectral resolution was 4 cm^−1^ with 64 scans with a range of 600 to 4000 cm^−1^.

### 4.7. Molecular Weight Determination

Viscosity average molecular weight was determined by viscosimetry. The viscosity measurements were performed using an Ubbelohde capillary viscometer (525-20 capillary) at 25.0 ± 0.1 °C and ViscoClock equipment (Fisher Scientific, Madrid, Spain). Samples were dissolved in 0.3 M AcOH/0.2 M AcNa. The average viscosity molar mass was calculated from the Mark Houwink equation (Equation (6)):

(6) $$\left[\eta \right]=k*Mv^{\alpha }$$

where *η* represents the intrinsic viscosity of the polymeric solution, *K* and *α* are constants that depend on the nature of the polymer and the solvent, and *Mv* is the average viscous molecular weight. Under our experimental conditions, *k* = 0.00076 and *α* = 0.76 when the intrinsic viscosity is expressed in dL/g [29].

### 4.8. Chromatography

GPC-HPLC was performed with a Waters 625 LC System pump with an Ultrahydrogel column (Waters, i.d. = 7.8 mm, l = 300 mm) thermostated at 35 °C. A Waters 2414 differential refractometer was used in the detection (Waters, Madrid, Spain). Ammonium acetate/0.2 mol/L acetic acid buffer (pH 4.5, 0.15 mol/L) was used as eluent. The flow rate was 0.6 mL/min and 20 μL samples dissolved in the buffer were injected.

### 4.9. XRD Studies

Samples were analyzed in a PANalytical model X’Pert MPD multi-purpose diffractometer (PANalytical B.V, Almelo, The Netherlands) with copper radiation at room temperature at an angular range (2θ angle) from 5° to 60°. Crystallinity indexes (*CrI*) were calculated from Equation (7) [59].

(7) $$CrI\;\left(\%\right)=\frac{I_{110}-Iam}{I_{110}}$$

where *I*_110_ is the maximum intensity of the reflection at 2θ = 20°, and *I*_am_ is the minimum intensity of the diffraction in the amorphous region at 2θ = 16°.

### 4.10. Thermal Analysis

Thermal analysis of the polymer samples was carried out in air. Thermogravimetric (TG) analysis and differential (DTG) thermogravimetric analysis were carried out with a TA Instruments SDT Q600 system (TA instruments, New Castle, DE, USA). Analyses were carried out with around 5 mg sample in aluminum pans under air (100 mL/min) between 20 and 800 °C. The experiments were run at a scanning rate of 10 °C/min.

### 4.11. Scanning Electron Microscopy (SEM)

The microstructure of the polymers was observed by scanning electron microscopy (SEM). The samples were examined using a scanning electron microscope (JEOL JSM-6335, JEOL, Tokyo, Japan) after being covered with Au.

### 4.12. Antioxidant Activity

The α,α-diphenyl-β-picrylhydrazyl DPPH radical-scavenging activity of the polymers was assayed by the method proposed by Chen et al. [60], with slight modifications. A total of 250 μL of the sample solution in 5 mg/mL acetic acid was mixed with 1 mL of methanolic DPPH solution (100 μmol/L). The mixture was shaken and kept at room temperature in the dark. After 60 min, the absorbance was measured at 517 nm. The control sample was a DPPH-methanol solution. The antioxidant activity was calculated from a Trolox (6-hydroxy-2,5,7,8-tetramethylchroman-2-carboxylic acid) standard curve and expressed in µM Trolox equivalent

### 4.13. Antimicrobial Activity

Antimicrobial activity of chitin and chitosan samples, both in power and in solution, was tested against *Escherichia coli* (Migula) Castellani and Chalmers (ATCC 11775) and *Staphylococcus aureus* subsp. aureus Rosenbach (ATCC 29213) as models for Gram-negative and Gram-positive bacteria. The assay was carried out following the macrodilution test by the Clinical and Laboratory Standards Institute [61]. Briefly, 2% w/v chitosan was initially dissolved in 0.15 mol/L ammonium acetate/0.2 mol/L acetic acid buffer (pH 4.5), and 0.5 mL of this solution was added to 1 mL of sterile LB media. After that, 1 mL of bacterial inoculum was added (1 × 10^6^ FCU/mL). This tube was used to start serial dilutions in LB media. For powder samples, chitin or chitosan were suspended in LB media at 10 mg/mL and the bacterial inoculum was added ((1 × 10^6^ UFC/mL). In both types of samples (powder and solution), tubes were incubated at 37 °C for 18 h and visually evaluated. MIC was defined as the minimum polymer concentration that completely inhibited the bacterial growth seen with the naked eye. In order to check that growth had been inhibited, 100 μL of each tube was inoculated in an agar plate with Muller Hinton media and the plates were incubated for 24 h at 37 °C. Each experiment was carried out in duplicate, and two crustacean chitin and chitosan samples from crab and lobster were also tested for comparative purposes. Negative controls without polymer and bacteria, and positive controls with bacteria and without polymer, were carried out.

## 5. Conclusions

*S. entomon* is an appropriate source for chitin and chitosan extraction with isolation rates in the range of other sources. Elementary analysis and FTIR-ATR spectra revealed the presence of impurities that must be studied in more detail in the future to determine their nature, and their possible effects on biological properties. Chitosan of medium molecular weight and with an acetylation degree of about 0.08 was isolated. This sample was easily handled in biological assays. Crystallinity and thermal properties revealed that isolated chitin and chitosan samples exhibited similar parameters to those of other crustacean sources. *S. entomon* chitosan exhibited greater antioxidant activity than other chitosan samples described in the literature, as well as antimicrobial activity against *S. aureus* but not against *E. coli*. This antioxidant activity and antimicrobial behavior indicates this organism is a source for chitin and chitosan to be applied in the biomedical field, in particular in applications in wound healing.

## Figures and Tables

**Table 1:** Elementary analysis of chitin and chitosan samples.

Sample	C, %	H, %	N,%	Ratio C/N
Chitin	47.25	6.61	6.41	7.37
Chitosan	48.30	6.67	6.58	7.34

**Table 2:** Antimicrobial activity of chitin and chitosan, from different sources, in powder form.

Source	Bacteria	Material	CFU Counted	% growth	Growth
***Saduria entomon***	*E. coli*	chitin (CH)	lawn	>99.9%	+
		chitosan (CHT)	lawn	>99.9%	+
	*S. aureus*	chitin (CH)	lawn	>99.9%	+
		chitosan (CHT)	lawn	>99.9%	+
**Crab**	*E. coli*	chitin (CH)	lawn	>99.9%	+
		chitosan (CHT)	lawn	>99.9%	+
	*S. aureus*	chitin (CH)	lawn	>99.9%	+
		chitosan (CHT)	lawn	>99.9%	+
**Lobster**	*E. coli*	chitin (CH)	lawn	>99.9%	+
		chitosan (CHT)	lawn	>99.9%	+
	*S. aureus*	chitin (CH)	lawn	>99.9%	+
		chitosan (CHT)	lawn	>99.9%	+

CFU: colony-forming unit.

**Table 3:** Antimicrobial assay for chitosan solutions.

Source	Bacteria	Chitosan Concentration, mg/mL	CFU Counted	Growth, %	Growth
***Saduria entomon***	*E. coli*	3.34	>500	99.9%	+
		1.11	lawn	>99.9%	+
		0.37	lawn	>99.9%	+
		0.125	lawn	>99.9%	+
	*S. aureus*	3.34	0	0%	-
		1.11	7	0.001%	-
		0.37	lawn	99.9%	+
		0.125	lawn	>99.9%	+
**Crab**	*E. coli*	1.5	lawn	>99.9%	+
		0.375	lawn	>99.9%	+
		0.1	lawn	99.9%	+
		0.02	lawn	>99.9%	+
	*S. aureus*	1.5	4	0.0004%	-
		0.375	0	0%	-
		0.1	lawn	>99.9%	+
		0.02	lawn	>99.9%	+
**Lobster**	*E. coli*	3.34	0	0%	-
		1.11	>500	99.9%	+
		0.37	lawn	>99.9%	+
		0.125	lawn	>99.9%	+
	*S. aureus*	3.34	>500	99.9%	+
		1.11	lawn	>99.9%	+
		0.37	lawn	>99.9%	+
		0.125	lawn	>99.9%	+

## Data Availability

Data available on request from the authors.
